# Pandemic-Driven Shifts in Epstein-Barr Virus (EBV) Epidemiology: Single Center Study

**DOI:** 10.3390/v17030375

**Published:** 2025-03-06

**Authors:** Maria Eugenia Amarillo, Karen Lindl, Mercedes García Lombardi, Maria Victoria Preciado, Elena De Matteo, Paola Chabay

**Affiliations:** 1 Molecular Biology Laboratory, Pathology Division, Multidisciplinary Institute for Investigation in Pediatric Pathologies (IMIPP), CONICET-GCBA, Ricardo Gutierrez Children’s Hospital, Ciudad Autónoma de Buenos Aires C1425EFD, Argentina; maria.eugenia.amarillo41@gmail.com (M.E.A.); karenlindl98@gmail.com (K.L.); preciado@conicet.gov.ar (M.V.P.); elenadematteo@gmail.com (E.D.M.); 2 Oncology Division, Ricardo Gutierrez Children ’s Hospital, Ciudad Autónoma de Buenos Aires C1425EFD, Argentina; dragarcialombardi@gmail.com

**Keywords:** EBV, pandemics, Hodgkin lymphoma, children

## Abstract

Social distancing, hand hygiene, mask wearing, surface decontamination, travel restrictions, and school closures have been implemented worldwide to control coronavirus disease 2019 (COVID-19). It was reported that the number of EBV infections as well as the age characteristics of infected persons before and after the COVID-19 pandemic significantly decreased in children from China. Since no studies have explored the changes in EBV-associated lymphomas so far, our aim was to explore EBV infection and viral-associated Hodgkin lymphoma (HL) in a pediatric cohort from a single center. A decrease in EBV+ children by serology was proved, in particular, in those undergoing primary infection, along with a significant increase in the mean age of healthy carriers. Furthermore, a decrease in EBV-associated pediatric cHL was observed post-pandemic, particularly in the NS subtype, with a marked decrease in cases diagnosed from 2022 onward. Even though the underlying reasons for the change in incidence rates seen in this study still remain speculative, it could be hypothesized that, after the pandemic, older children have a better ability to control the EBV-mediated lymphomagenesis, based on the fact that the age of infected patients increased.

## 1. Introduction

Epstein-Barr virus (EBV) is a ubiquitous γ-herpesvirus that infects more than 90% of adults globally. It spreads among individuals through saliva, reaching the tonsil, the site of primary infection and reactivation, where EBV infects B cells, its main target cell [1]. Primary infection is typically acquired orally during childhood or adolescence, but the age of primary infection has been gradually increasing over time in developed countries [2]. In contrast, in populations with lower socio-economic status, children may be more likely to become EBV carriers at an earlier age [3].

Since its discovery in 1964, EBV was found to be linked with a broad range of cancers. In 1997, EBV was classified as a group 1 carcinogen by the International Agency for Research on Cancer (IARC) because of its causal association with Endemic Burkitt lymphoma (eBL), Hodgkin lymphoma (HL) and Nasopharyngeal carcinoma (NPC) [4]. Thereafter, EBV was also linked to other cancers such as Extranodal NK/T-cell lymphoma, Nasal type, (ENKTLNT), Diffuse large B-cell lymphoma (DLBCL) and Gastric Carcinoma (GC), in addition to other non-malignant diseases such as multiple sclerosis (MS) [5]. In 2010, EBV-associated cancers caused 1.8% of all cancer related deaths worldwide, a number which had increased by 14.6% since 1990 [6]. More recent data from 2020 indicate that the six major EBV-associated malignancies, NPC, GC, HL, BL, DLBCL, and NK/T cell lymphoma, accounted for 239,700–357,900 new cases and 137,900–208,700 deaths, indicating the extensive impact EBV has on global health [7].

Classical HL (cHL) is subclassified into nodular sclerosis (NS), mixed cellularity (MC), lymphocyte-rich (LRHL), and lymphocyte-depleted (LDHL) [8]. CHL includes three different disease entities: pediatric HL (EBV-positive, mixed cellularity subtype), HL in young adults (EBV-negative, nodular sclerosis subtype), and HL in older adults (EBV-positive, mixed cellularity subtype) [9]. The MC histological subtype was demonstrated to be mostly associated with EBV and prevail in young children, while the NS subtype is mostly seen in adolescents and young adults and comprises about 75% of HL. CHL tumor cells are EBV positive in ~30 and 90% of all pediatric cases in developed and underdeveloped or developing countries, respectively [10].

In Argentina, EBV primary infection shows the classical pattern observed in developing populations, given that nearly 70% of patients are seropositive by the age of 2 years, while a statistically significant EBV association with children ≤10 years old was demonstrated in B-cell lymphomas, suggesting a relationship between low age of EBV seroconversion and B-cell lymphoma development risk [11]. In a previous study in a single center published by our group, EBV association with pediatric cHL was analyzed for a 28-year period. Remarkably, a slight bias of EBV association with the nodular sclerosis (NS) subtype was observed, whereas even though MC still prevailed in the whole series, those cases diagnosed as NS showed a sustained rise [12].

Social distancing, hand hygiene, mask wearing, surface decontamination, travel restrictions, and school closures have been implemented worldwide to control coronavirus disease 2019 (COVID-19). Those interventions have been demonstrated to reduce the rate of viral agent detection for lower respiratory and gastrointestinal infections [13]. In relation to EBV, the number of EBV infections as well as the age characteristics of infected persons in four years before and after the COVID-19 pandemic significantly decreased in children from Henan, China [14]. Furthermore, the total number of seropositive EBV infections in 2020 was reduced by 30% compared to that in 2019 in children from Zhejiang, China [15]. In contrast, as far as we know, no studies have explored the changes in EBV-associated lymphomas. Therefore, the aim of this study was to explore EBV infection and viral-associated HL in a pediatric cohort from a single center, in order to disclose the influence of social restrictions on EBV epidemiology.

## 2. Materials and Methods

### 2.1. Patients and Samples

Formalin-fixed paraffin-embedded (FFPE) biopsy samples were obtained from 112 patients diagnosed with cHL according to the WHO criteria [16]. The samples were collected from the archives at Pathology Division, Ricardo Gutierrez Children’s Hospital, a national referent institution that receives cases from the whole country, from 2010 until 2024. Some of the samples collected from 2010 until 2018 were included in previous studies [12]. In addition, 106 serum samples collected from 2016 until 2024 from children undergoing tonsillectomy due to nonreactive hyperplasia diagnosed according to international routine protocols for recurrent chronic inflammation at the Otorhinolaryngology Division, Ricardo Gutierrez Children’s Hospital (Buenos Aires, Argentina) were used in this study. Some of the samples collected from 2016 until 2019 were included in previous studies [17]. The year 2020 was defined as the cut off for the pre and post pandemic periods.

The Ethics Committee of the Ricardo Gutiérrez Children’s Hospital approved the study protocol under the number CEI n° 20.37, and gave their supervision. All samples were collected after written informed consent (for patients older than 12 years old and legal guardians of children younger than 12 years old), and assent (7- to 12-year-old patients and legal guardians of children older than 12 years), following the national and international ethics standards and under the supervision of the Ethics Committee of the Ricardo Gutiérrez Children’s Hospital, in accordance with the Helsinki Declaration of 1975.

### 2.2. EBV Analysis

EBERs in situ hybridization (ISH) was performed on the 112 FFPE cHL tissue sections using fluorescein isothiocyanate (FITC)- conjugated EBERs oligonucleotides as probes (Dako) according to the manufacturer’s instructions. A monoclonal antibody, anti-FITC labeled with alkaline phosphatase, was used for the detection of hybridized sites (Dako). An EBV-associated post-transplant lymphoproliferative disorder was used as a positive control.

EBV serology was performed on 106 serum samples by indirect immunofluorescence (IFI) to determine the presence of serum antibodies: VCA-IgG, VCA-IgM, and EBNA1-IgG. Patients with primary infection (PI) were defined by the presence of VCA-IgM and VCA-IgG antibodies; healthy carrier patients (HC) by the presence of VCA-IgG and EBNA1-IgG antibodies; and non-infected patients (NI) by the absence of EBV antibodies.

### 2.3. Statistical Analysis

Categorical variables (EBV presence, age group, gender, HL subtypes) were analyzed using Fisher’s exact test. Mann–Whitney or *t* test was used to compare the age means in relation to EBV presence and histological subtypes. The Mantel–Haenszel test was used to compare the frequencies between groups while controlling potential confounding factors, providing a stratified analysis of the association between categorical variables. All tests were two-tailed, and a *p* < 0.05 was considered statistically significant.

## 3. Results

### 3.1. EBV Prevalence in Children and in cHL

EBV association with pediatric cHL was observed in 68/112 patients (60.7%) by EBERs ISH. As expected, EBV was statistically associated with the MC subtype since it was observed in 74% of cases, compared to 48% in NS (*p* = 0.0232, Fisher exact test) in the overall series. Additionally, EBV presence was statistically associated with patients younger than 10 years old (70.6% of EBV+ cases) (*p* = 0.0302, Fisher exact test). In order to compare with EBV prevalence in children, serology was assessed to discriminate between patients with primary infection (PI), healthy carriers (HC), and non-infected children. Among 106 patients, 31/106 (29.2%) were PI, 70/106 (66%) were HC, and the remaining 11/106 (10.4%) were NI.

**Figure 1:**
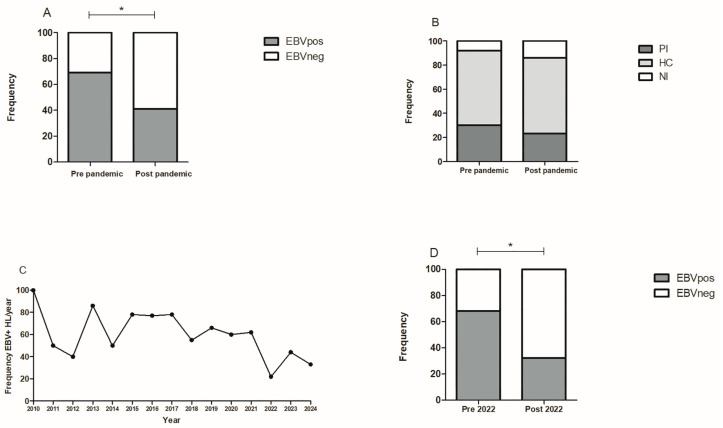
Frequency of EBV+ cases. (**A**) Frequency of EBV+ HL cases by EBERs ISH in children before and after the pandemic. (**B**) Frequency of EBV+ cases by serology before and after the pandemic. (**C**) Frequency of EBV+ HL cases per year by EBERs ISH from 2010 until 2024. (**D**) Frequency of EBV+ HL cases by EBERs ISH before and after 2022. * *p* < 0.05.

With the purpose of evaluating the effect of pandemic restrictions on EBV association with pediatric lymphoma, in comparison with its distribution in the pediatric population, cases diagnosed before and after the pandemic were differentiated. Among the 112 cHL pediatric patients, 78 were diagnosed before and 34 after the pandemic, and 54/78 (69.2%) and 14/34 (41.2%) were EBV+, respectively (Figure 1A). The percentage change in EBV-associated pediatric cHL between both periods was evaluated as %EBV+ cHL after the pandemic %EBV+ cHL before the pandemic/%EBV+ cHL before the pandemic ×100. This revealed a 40.5% decrease in EBV-associated pediatric cHL after the pandemic. Regarding EBV-infected children, the decrease in EBV infection was 7.05% after the pandemic, since 71/77 (92.2%) children were seropositive for EBV before, and 30/35 (85.7%) after. Remarkably, when EBV infection was discriminated, 23/77 (29.9%) were PI before the pandemic, compared to 8/35 (22.9%) after, indicating a 23.4% decrease in primary EBV infection. In line with this, 6/77 (7.8%) were NI before the pandemic, while 5/35 (14.3%) were NI after, showing a decrease of 23.4% (Figure 1B). Although no statistical differences were observed in the frequencies of PI, HC, and NI children before and after the pandemic (*p* > 0.05), a statistically significant decrease in EBV-associated pediatric cHL was observed post-pandemic (*p* = 0.006, Fisher’s exact test). When this decrease was further stratified considering histological subtypes, the NS subtype after the pandemic showed a significant reduction in EBV+ pediatric cHL (*p* = 0.025, Mantel–Haenszel test), despite no significant differences in subtype distribution between the two periods (*p* > 0.05, Fisher’s exact test). The decline in EBV+ cases was especially notable in 2022 (Figure 1C). A deeper analysis of the data before and after 2022 further confirmed this significant reduction in EBV+ cases starting from that year (*p* = 0.0029, Fisher’s exact test) (Figure 1D).

### 3.2. EBV Distribution According to Age

**Figure 2:**
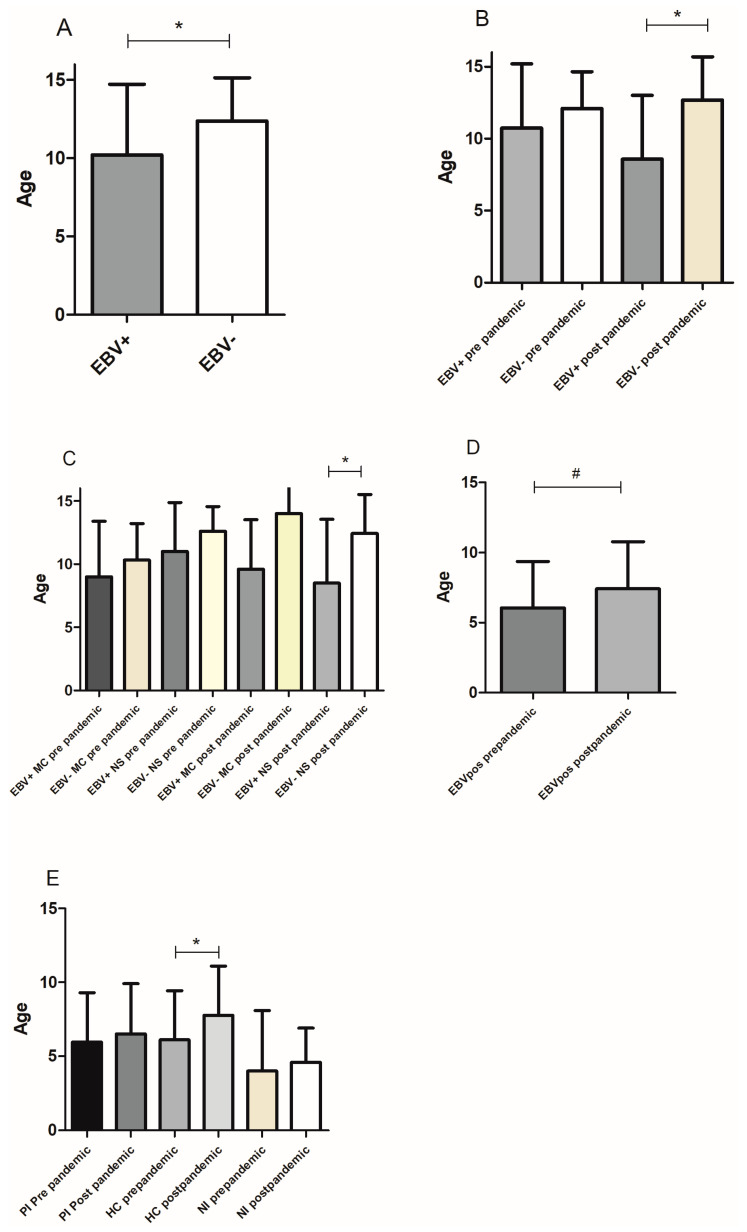
Age distribution in relation to EBV infection. (**A**) Mean age of EBV+ vs. EBV− cHL cases in the entire cohort. (**B**) Mean age of EBV+ vs. EBV− cHL cases before and after the pandemic. (**C**) Mean age of EBV+ vs. EBV− cHL cases specifically in MC and NS subtypes before and after the pandemic. (**D**) Mean age of EBV+ cases by serology in children before and after the pandemic. (**E**) Mean age of EBV serological status before and after the pandemic. * *p* < 0.05; # trend.

When analyzing cHL distribution according to age, as expected, mean age in EBV+ cHL in the whole cohort was statistically lower than in its EBV− counterpart (*p* = 0.0444, Mann–Whitney test) (Figure 2A). This difference was even more pronounced in cases diagnosed after the pandemic (*p* = 0.0145, Mann–Whitney test) (Figure 2B). Among the most prevalent subtypes, MC and NS, EBV+ patients were younger than EBV− ones in all subtypes, but statistical significance was only observed for the NS subtype after the pandemic (*p* = 0.0365, unpaired *t* test) (Figure 2C), whereas no differences were observed in the mean age between other cHL subtypes (*p* > 0.05, Mann–Whitney test). To compare the effects of social restrictions on the age of EBV infection, the mean age of EBV-infected children before and after the pandemic was evaluated, showing a trend toward a younger age before the pandemic (*p* = 0.0578, Mann–Whitney test) (Figure 2D). This difference became significant particularly in HC children before the pandemic (*p* = 0.0480, Mann–Whitney test) (Figure 2E).

## 4. Discussion

The measures for preventing and controlling SARS-CoV-2 infection have significantly impacted people’s lifestyles and the transmission of many other pathogens. Several studies have indicated that the incidence of febrile respiratory viral infections, including influenza and respiratory syncytial virus, as well as enteric viral infections like adenovirus, norovirus, and rotavirus, have significantly decreased during the COVID-19 pandemic [18,19,20]. In fact, following the initiation of local COVID-19 restrictions in week 14, influenza and RSV activity declined and remained very low relative to previous seasons, suggesting that changes in health-seeking behavior in the setting of COVID-19 restrictions may have contributed to those reductions [18]. Regarding herpesvirus infections, the incidence rates and time of CMV infection were affected by COVID-19 [21]. One of the defining features of herpesviruses is their ability to transition from a latent state to lytic reactivation. In fact, in COVID-19 patients, detectable Kaposi Sarcoma virus load was associated with death after adjusting for age, sex and HIV status, suggesting that SARS-CoV-2 infection may cause reactivation of KSHV in latently infected individuals [22].

EBV is a herpesvirus prevalent throughout the world population. Before the pandemic, some studies reported epidemiological changes in EBV infection, like in France [23]. Some previous research has demonstrated EBV lytic reactivation, confirmed by the increase in viral load, following SARS-CoV-2 infection [24], while other studies have not confirmed an increase in EBV load [22]. When serological studies were conducted, the total number of seropositive EBV infections as well as primary infection in 2020 in Zhejiang, China, showed a 30% reduction compared to 2019. The trends in seropositive rates of age distribution between 2019 and 2021 were similar in this population, but the seropositive rates of children aged 6 months–6 years in 2020 were lower than those in 2019. Furthermore, in children aged 1–3 years, seropositivity dropped by 40% compared to 2019. This study proposes that this observation may be explained by the fact that children had relatively less contact with the saliva of people infected with EBV after the COVID-19 pandemic [15]. In line with this, in children from Henan, China, after the COVID-19 pandemic, the children seropositive for EBV IgM (as a marker of PI) and EBV IgG (as a marker of HC) decreased in all age ranges, indicating that the COVID-19 pandemic reduced the infection of EBV in children in this population, suggesting a relation with the strict measures taken during the COVID-19 pandemic, such as suspension of classes, increased awareness of wearing masks, and paying attention to hand hygiene [14]. In line with these findings, in our series from a single institution, even though the difference was not significant, a decrease in EBV+ children by serology was proved, particularly in those undergoing primary infection. As expected, this decrease was followed by a corresponding increase in the percentage of uninfected children, suggesting that the COVID-19 prevention and control measures may have played a role in containing EBV infection. This control perhaps also involved a shift in the age of infection, given that EBV-infected children were older after the pandemic, in particular, HC. EBV in underdeveloped populations like Argentina is transmitted by exposure to infected saliva, either directly or via, for example, unclean toys or fingers, that may explain differences related to the socioeconomic condition among transmissions. The measures to control COVID-19 included prevention measures such as hand washing with soap or alcohol-based hand gel, disinfecting surfaces, and social distancing. Therefore, these measures probably played a major role in EBV epidemiological changes, such as the shift to older age, when uninfected children came into contact again—when social control measures were relaxed—with a larger number of people infected with EBV.

In addition, it can be hypothesized that in older children, the immune system is more mature and has an increased ability to control complications arising from viral infection. In Argentina, both EBV infection and the incidence of virus-associated lymphomas occur in children at early age. Furthermore, our group previously observed more active virus infection in the tonsils of pediatric HC, such as the expression of latency III and lytic antigens [25], which could lead to complications due to less restrictive expression of viral oncogenes. If this active infection occurs at older ages, in the context of a more mature immune system, it is plausible that these children will have a better capacity to control the oncogenic process that triggers EBV-associated lymphomas. In children with primary EBV infection, a decrease in the magnitude of peripheral CD8+ T cell responses to EBV lytic peptides, in contrast to an increase in their response to latent peptides, was shown compared to adults [26]. NK cell responses may play a key role in combating EBV infection early in life in children, in particular, to attenuate IM symptoms [27]. Considering the NK cell population in children, a significant variation within several NK cell markers not only among adults but also in the pediatric population across different ages was described. Furthermore, upregulation of CD69 activation marker showed a trend toward lower expression in the younger children [28]. Those observations may reinforce the notion of a greater capacity to control EBV infection in older children.

In Germany, the incidence of childhood cancer in 2020, when compared to data from the previous five years, revealed similar or even higher monthly rates of new pediatric cancer diagnoses than those observed between 2015 and 2019. Furthermore, the estimated age-standardized incidence rates were significantly higher in 2020 across all diagnostic groups compared to the rates from 2015 to 2019 [29]. Specifically, in patients with lymphoma, the impact of the COVID-19 pandemic in Sweden showed a consistently lower number of patients diagnosed with lymphoma from the onset of the pandemic in March–June 2020, primarily driven by a lower number of diagnosed indolent lymphomas, whereas the number of patients diagnosed with aggressive lymphomas remained similar. Then, incidence rates were similar to those in 2019, but slightly altered patient characteristics with a higher median age [30]. In Argentina, the Global Cancer Observatory (IARC), in its 2022 report, indicated prevalence of HL in the last 5 years of 7.1 per 100,000 cases. However, there is no detailed information on pediatric cases, nor comparative prevalence data from before and after the pandemic [31]. Regarding EBV-associated lymphomas, no previous studies explore the differences before and after the pandemic. In our series, a decrease in EBV-associated pediatric cHL was observed post-pandemic, particularly in the NS subtype, with a marked decrease in cases diagnosed from 2022 onward. A decline in EBV-associated pediatric cHL over the past decades was also observed in 155 children from Brazil, from 96% to 64%, and in 46 children from Korea, from 21% to 8.6% [32,33]. Improved socioeconomic conditions in these two populations have been suggested as a factor contributing to this decline [31]. This trend may be associated with the post-pandemic decrease in EBV-associated cHL cases observed in our series. Our group previously reported a shift from the MC to the NS subtype prior to the pandemic, along with an increase in EBV association with the NS subtype, the prevalent subtype in more developed populations [12]. It seems that after the pandemic, this trend reversed, with a decrease in the association of EBV with the NS subtype, but without a corresponding increase in EBV associated with the MC subtype. Therefore, the decline in EBV-associated cHL after the pandemic can be attributed specifically to this decrease in the NS subtype. Although this concept is speculative and should be confirmed over time with a study on a larger sample, the decrease in NS subtype, regardless of EBV infection, observed after the pandemic may be due to certain specific changes that worsen socioeconomic conditions. Given that in this subtype, only younger patients were susceptible to EBV-associated cHL, the observed shift in EBV infection to older children post-pandemic suggests a potential role of immune maturation in controlling EBV-mediated lymphomagenesis, though further studies are needed to confirm this mechanism.

In summary, this study provides some insight into the decrease in EBV-associated pediatric HL in a population from an underdeveloped country. The underlying reasons for the change in incidence rates seen in this study still remain speculative. Its most important weakness is the number of patients, since it represents a preliminary observation conducted in a single institution, where the sample size is relatively small. Therefore, considering the small number of participants, the findings must be interpreted carefully, but the observed trends suggest potential associations that warrant further investigation. It may be essential to enlarge the sample size and follow up on this study in the coming years to assess whether this trend reverses and returns to pre-pandemic levels of EBV-associated pediatric cHL. Moreover, additional studies in other populations with a larger number of patients, such as multicenter studies within Latin America or longitudinal analyses of post-pandemic birth cohorts to track EBV infection age shifts, are required to confirm these findings. Continued close monitoring of incidence patterns may shed light on the underlying reasons for these findings and contribute to understanding disease etiology.

## Data Availability

The datasets generated during and/or analyzed during the current study are contained within the article.

## Abbreviations

The following abbreviations are used in this manuscript:

EBV	Epstein Barr Virus
HL	Hodgkin Lymphoma
IARC	International Agency for Research on Cancer
eBL	Endemic Burkitt lymphoma
NPC	Nasopharyngeal carcinoma
ENKTLNT	Extranodal NK/T-cell lymphoma, Nasal type
DLBCL	Diffuse large B-cell lymphoma
GC	Gastric Carcinoma
MS	Multiple Sclerosis
NS	Nodular Sclerosis
MC	Mixed Cellularity
FITC	Fluorescein isothiocyanate
PI	Primary Infection
HC	Healthy Carriers
NI	Non-Infected
CMV	Cytomegalovirus
HIV	Human Immunodeficiency Virus
KSHV	Kaposi Sarcoma Herpes Virus
